# Telemedicine in Rheumatoid Arthritis: A Review of the PubMed Literature

**DOI:** 10.31138/mjr.34.1.16

**Published:** 2023-03-31

**Authors:** Nur Barlas, Sait Berk Barlas, Shristi Basnyat, Emre Adalier

**Affiliations:** 1 Department of Internal Medicine, Cape Coral Hospital, Cape Coral, Florida, United States,; 2 Department of Rheumatology, Cape Coral Hospital, Cape Coral, Florida, United States,; 3 University of Pavia Faculty of Medicine and Surgery Ringgold Standard Institution, Pavia, Lombardia, Italy

**Keywords:** telemedicine, telehealth, rheumatoid arthritis

## Abstract

**Objective:**

The COVID-19 pandemic increased the use of telemedicine in the management of rheumatoid arthritis (RA) patients. The paper intends to provide a narrative review of the PubMed literature (2017–2023) on the application of telemedicine in the management of RA to identify the trends in the application of telemedicine in RA and future research needs.

**Methods:**

The PubMed database was used to research data. “Telemedicine” and “rheumatoid arthritis” keywords were entered in the search box. Out of 126 publications between 2017–2023, ones that did not directly address RA, not relate to telemedicine, case reports, preliminary reports and comments to editors were screened. 31 articles were selected for the study.

**Results:**

27 of 31 studies support the value of telemedicine in the monitoring of RA patients. Patient-reported outcomes mostly report positive perceptions, high satisfaction, and convenience. There was no statistically significant difference between telemedicine and hospital visits. Four studies reported the quality of care of telemedicine consultations was inferior to in-person consultations. One of these four studies reported limited health and digital literacy, and older age reduce satisfaction with telemedicine. Comparative and randomised clinical studies and research on modes of telemedicine were limited in quantity. Limitations in study design, lack of evaluation in various settings may impact the generalisability of findings.

**Conclusion:**

This review suggests that telemedicine is beneficial in the management of RA, however more studies are needed to pinpoint the most effective uses of telemedicine and to explore alternative health care services for patients with barriers to access telemedicine.

## INTRODUCTION

Rheumatoid Arthritis (RA), a chronic autoimmune disorder and inflammatory disease with varying symptoms, requires routine periodic visits to a rheumatologist.^1^ Close follow-up is crucial in patients with rheumatoid arthritis (RA) regarding their ongoing concerns and needs.^2^ The COVID-19 pandemic increased the assimilation of telemedicine in the management of rheumatoid arthritis patients and their disease follow-up.^3^ Although the concept of telemedicine is not new, it has escalated with the Covid pandemic and is becoming widespread in the management and monitoring of disease activity in RA.^4,5,6^ Telemedicine provides avenues through the help of technology to maintain receiving medical care at a physical distance from the healthcare provider.^5^ The American Medical Association (AMA) considers Telemedicine to be a subdivision of Telehealth and refers to it as the use of telecommunication technologies to provide health care services remotely across geographic barriers.^7^ The World Health Organization (WHO) addresses that “telemedicine” and “telehealth” terms “have been separately defined over time but are often used interchangeably with considerable overlap in scope”.^8^ Based on WHO Guideline: Recommendations on Digital Interventions for Health System Strengthening telemedicine is “the provision of healthcare services at a distance with communication conducted between healthcare providers seeking clinical guidance and support from other healthcare providers (provider-to-provider telemedicine) or conducted between remote healthcare users seeking health services and healthcare providers (client-to-provider telemedicine).”^9^ WHO 2019 guidelines explained that massive progress in telecommunications that include mobile devices, internet technologies, video-conferencing and mobile applications rapidly facilitated telemedicine implementation.^9^ Summing up WHO and AMA definitions telemedicine is receiving medical care by electronic information and telecommunication technologies and services that include remote patient monitoring, electronic medical record platforms, electronic apps, video conferencing, online interactions through a patient-provider portal when a patient and provider are distant.^7,8,9^ Based on this definition, tele-rheumatology is the use of telemedicine for the diagnosis and management of inflammatory and/or autoimmune rheumatic disease.^10^

## METHODS

This narrative literature review aims to provide a qualitative summary and overview of the current state of research on telemedicine in rheumatoid arthritis (RA) and identify future research needs. Instead of focusing on a specific clinical question and using analytical methods to collect, group and analyse data as in the systematic review,^11^ we intended to review the status of telemedicine in RA as broad in scope; highlight key findings and provide a subjective summary.^12,13^ The PubMed database, “the oldest and most popular hub of updated information on scholarly biomedical journals”^11^ was used to research data. Telemedicine and rheumatoid arthritis keywords were entered in the search box. 126 articles were identified between 2004–2022. Limiting the research to the last five years between 2017–2022 resulted in 93 publications. Then we extended the time frame of the review and searched 2017- February 1, 2023, on the PubMed database and included the most recent studies as well. Finally, out of a total 126 publications between 2017–2023, the publications that did not address RA, did not specifically relate to telemedicine, expert opinions, case reports, preliminary reports, and comments to editors were screened. 31 articles that seem relevant and valuable to the present research were selected for the study. The results of the review articles are outlined below.

## RESULTS

A study to determine the criterion associated with the use of telemedicine for RA collected data from 122 RA patients who were enrolled at the Alaska Native Medical Centre in Anchorage, Alaska between August 2016 and March 2018. RA patients filled out a telemedicine perception and Routine Assessment of Patient Index Data 3 (RAPID3) survey. The data was collected in 12 months. The study found that video telemedicine may be a viable alternative for medical care for rheumatoid arthritis (RA) patients who have high disease activity and have positive perceptions of telemedicine, and for rheumatologists who regularly use telemedicine. Demographic, comorbidities, or other factors were reported as insignificant in the telemedicine use of RA patients. The study also reported that the observational nature and the unique setting of the research may be prone to bias and confounding, and therefore findings may limit the generalization of results. The study suggested more future comparative studies on telemedicine vs usual care that investigate disease activity over time and the quality of care for RA.^14^

An interrupted time series analysis was applied to compare the e-Health platform usage of self-management outpatient clinic patients with RA between 2014–2019. It was found that the implementation of an e-Health platform had a positive effect and resulted in a significant decrease in outpatient clinic visits. Findings showed that on average, the implementation of remote patient monitoring resulted in 0.955 fewer visits per patient per year while maintaining disease control. The study concluded that using telemedicine in the management of RA is promising.^15^

120 RA patients were studied to determine the feasibility and performance of asynchronous telemedicine visits in four German university centres. RA patients using the medical app, ABATON, electronically reported the results of a self-performed quick CRP test, joint count, and self-reported outcomes in-between visits. It is found that rheumatologists’ treatment decisions were in common for both the asynchronous telemedicine RA patient-generated data and traditional in-person rheumatology clinic data. It is concluded that the study provides support for the value of implementing Telemedicine in the treatment of rheumatology.^16^

In a cross-sectional, multicentre study on 575 RA patients in 5 university hospitals in France, patients filled out a self-questionnaire collected to identify their use of eHealth tools. The study evaluated RA patients’ access, support, frequency of use, type of use, and reason for use of eHealth. It was found that 28.7% of RA patients used eHealth specifically for RA-related reasons. 66.4% of RA patients used eHealth to help monitor RA. 12.8% of RA patients used it for treatment reminders. 21.6% of the patients using eHealth also used a specific app for RA. Based on a multivariate analysis some factors such as membership of a patient association, use of biologic disease-modifying antirheumatic drugs, comorbidities, doctor recommendation, ease of use, and data security were found positively related to RA patients’ reliance on eHealth use. Based on univariate analysis, age, education level, employment status, treatment, comorbidities, membership of a patient association, and patient education program were associated with eHealth use for RA. The study concluded that although few RA patients have used eHealth for disease management, rheumatologists could encourage the use of a dependable and valid eHealth tool to optimize treatment management.^17^

A study that includes smartphone-based telemedicine was conducted on the estimation of the cost-effectiveness of Disease-Modifying Anti-Rheumatic Drugs (DMARDs) combined with regular disease monitoring therapy versus conventional monitoring of RA patients. The evaluation was based on 89 patients. Patients were split into two groups (conventional monitoring vs connecting monitoring). 45 patients with a connected monitoring interface on a smartphone were included in DMARDs therapy. 44 patients in the control group were conventionally monitored. For each group of patients’ health outcomes (the gain in quality-adjusted life-years-QALYs); economic analysis (collected data on unit costs) was measured. Patients receiving connected monitoring resulted in a significant cost reduction of 72€ and a slightly greater but not significant gain in the average QALY of 0.07; compared to conventionally monitored patients’ results. The study suggested that initiating a DMARD treatment using connected monitoring is more efficient and less expensive than conventional care. The study also suggested that the application of telemedicine in the intense monitoring of RA patients was beneficial.^18^

A study on the evaluation of the incidence of depressive disorders, anxiety, and fibromyalgia (FM) in RA patients during the COVID-19 pandemic suggested that the use of telemedicine in the clinical management of RA patients was helpful. The study concluded that telemedicine helped researchers recognize patients who needed in-person conversation for therapeutic re-evaluation. It is suggested that telemedicine will continue playing an important role in the management of rheumatology.^19^

Another systematic review on the effects of web-based health rehabilitation interventions for patients with rheumatoid arthritis investigated six studies from four trials (n = 567). The review revealed that although no adverse effects on pain, function, quality of life, self-efficacy, rheumatoid arthritis knowledge, and physical activity are reported, still findings are uncertain because of the limited evidence from small single trials. The review concluded that large trials are needed to better evaluate the effects of web-based rehabilitation interventions in patients with rheumatoid arthritis.^20^

A retrospective study analysed 431 RA patients during the Covid-19 pandemic on the use of telemedicine feasibility and impact on patient-reported outcomes revealed that telemedicine is beneficial and preferred by RA patients. Patients used the digital platform to enter data.

Researchers compared the general health outcomes of patients on telemedicine (35.3) to those who had hospital visits (39.3) and found no statistically significant difference in the results. There was also no statistically significant difference in the visual analog scale outcome of RA patients on telemedicine (33.3) and those who had clinic visits (37.1).^21^

A systematic review of the use of telemedicine on patient satisfaction, disease activity, and quality of life of 36 rheumatology reports published between 2015 and 2022 included 9 RA cases. The review reported that the use of telemedicine was effective, feasible and contributed to high levels of satisfaction. The review also concluded that due to the study design and risk of bias of previous studies randomized clinical studies will be necessary to better understand the use of telemedicine in rheumatology care.^22^

A systematic review and meta-analysis study of fifteen articles on the use of telemedicine applications in patients with chronic diseases reported that telemedicine is beneficial for enhancing the health of RA patients. In the systematic review telemedicine consultation and telemonitoring are found to be the most used intervention methods. The meta-analysis results showed that in the 6 months of intervention systolic blood pressure (MD = − 6.71; 95% CI = − 11.40, − 2.02; Z = 2.81; P = 0.005) was reduced and in the 12 months of intervention patients’ index of glycosylated haemoglobin (HbA1c) demonstrated improvement (MD = − 0.84; 95% CI = − 1.53, − 0.16; Z = 2.42; P = 0.02) In the review it was found that the use of telemedicine improved medication adherence in rheumatoid arthritis patients.^23^

In a qualitative study of seven focus groups of thirty-one RA patients, patient perspectives on electronic communication were researched during 2014–2015. The Andersen-Newman framework was applied in the analysis and the topic guide. The study identified electronic communication as an electronic recording of between-visit disease activity and sharing this information with healthcare providers or peers. The study found that RA patients were interested in electronic communication assistance in tracking disease activity, sharing data with health care providers electronically and having access to information about RA.^24^

On a different note, a study aimed to assess the perceived quality of life in rheumatoid arthritis (RA) patients during covid pandemic researched the role of telemedicine in the loneliness and satisfaction of RA patients. The De Jong-Gierveld Loneliness Scale (DJGLS), The Ankylosing Spondylitis Quality of Life Questionnaire (ASQoL), and the questionnaire of satisfaction with teleconsultations were given to one hundred and forty-three RA patients. The correlation analysis reported that loneliness (rho=0.1; p=0.01) was a significant (p <0.05) Independent predictor of QoL. There was a statistically significant inverse relationship between the level of loneliness and QoL. The results showed that teleconsultation visits were not helpful and were perceived negatively by patients. There was a statistically significant negative relationship between satisfaction with teleconsultations and perceived QoL.^25^

A study was conducted to provide information on functional status measures for telehealth visits and adaptation of American College of Rheumatology (ACR) rheumatoid arthritis disease activity. The study panel looked at the implementation of the Clinical Disease Activity Index [CDAI], Disease Activity Score in 28 joints using the erythrocyte sedimentation rate or the C-reactive protein level [DAS28-ESR/CRP], Patient Activity Scale II [PASII], Simplified Disease Activity Index [SDAI], and Routine Assessment of Patient Index Data 3 [RAPID3]) and functional status (the Health Assessment Questionnaire II [HAQ-II], Multidimensional Health Assessment Questionnaire [MDHAQ], and PROMIS physical function [PROMIS PF-10]) measures in telehealth settings. The study found that disease activity: PAS-II, RAPID3; functional status: HAQ-II, MDHAQ, PROMIS PF-10) requires minimal alteration for use in telemedicine. The study reported that the CDAI, DAS28-ESR/CRP, and SDAI measures can be determined based on patient-reported swollen and tender joint counts. The study concluded that the use of telehealth, such as electronic health record collection, and mobile applications, facilitate the ACR-recommended RA disease activity and functional status measures and support high-quality clinical care. The study also suggested further research to pinpoint the role of telehealth on the validity of these measures.^26^

An online survey was conducted to assess the role of the COVID-19 lockdown on stress vulnerability, resilience, and mood disturbances in fibromyalgia (FM) and rheumatoid arthritis (RA) patients through a telemedicine approach. Anonymous survey data were collected on disease activity, psychometric scales, and Zung Anxiety and Depression Self-assessment Scale from FM, RA, and healthy controls sample. The survey findings showed that during the lockdown the disease severity was higher in RA patients compared to FM patients based on the respective historical cohorts. Yet RA patients showed greater resilience and lower stress, anxiety, and depression scores compared to fibromyalgia patients. The study suggested that using telemedicine to screen for severe symptoms was beneficial to the management of rheumatic patients.^27^

A prospective pilot study was conducted to evaluate the impact of tele-rheumatology on RA patients in Iran during the COVID-19 Pandemic during March–June 2020. 122 rheumatoid arthritis patients who enrolled in telemedicine services were given a Health Assessment Questionnaire and followed by phone interviews to assess patient-reported satisfaction with teleconsultations. The study found that more than three-quarters of RA patients found the telemedicine service beneficial and efficient in terms of saving time and money and were inclined for using and recommending this service in the future. The study concluded that the use of telemedicine was helpful in the management of rheumatology during the pandemic.^28^

Interestingly, a retrospective analysis was performed to compare the quality-of-care and medical records regulation compliance of in-person consultations and telemedicine consultations on rheumatoid arthritis management during the COVID-19 pandemic. 324 medical notes, 208 in-person consultation notes, and 114 telemedicine consultation notes, related to rheumatoid arthritis consultations between July and December 2020 were reviewed and abstracted. It was found that the quality of care of in-person consultations was significantly superior to telemedicine consultations, 60% vs. 50%. Compliance with medical records regulations was similar between both groups. Findings showed that telemedicine consultations were a significant risk factor for no treatment changes, OR 2.113. The study concluded that the quality of care of telemedicine consultations is inferior to in-person consultations and impacts the management of rheumatoid arthritis.^29^

A randomised control trial on 44 early rheumatoid arthritis patients compared telemonitoring with an intensive treatment strategy and the conventional management strategy. They developed a website platform to perform remote telemonitoring. Based on the levels of improvement of the RA Impact of Disease (RAID) scores, the patient received pre-programmed advice and clinical case managers were notified. Depending on the situation attending physicians were notified and the patients were encouraged to visit the clinic for follow-up and treatment modification. The study evaluated remission rates, clinical disease activity index (CDAI), time to achieve remission, functional impairment, and radiological damage progression. It was found that 38.1% of patients in the telemonitoring group and 25% in the conventional management group achieved CDAI remission at year 1.

The time to achieve remission was significantly shorter in the telemonitoring group, 20 weeks vs. 36 weeks. Also, the patients in the telemonitoring group showed significantly greater improvement in functional impairment and radiological damage progression. The study concluded that the use of telemonitoring with an intensive treatment strategy for early rheumatoid arthritis patients leads to faster comprehensive disease control and higher remission rates than conventional management strategies.^30^

A systematic review of the effectiveness of telemedicine compared to the standard of care regarding disease activity, quality of life, functional activity, and patient satisfaction was performed. The review included four randomised control trials, one cross-out study, and five systematic reviews regarding rheumatoid arthritis patients and SLE patients. It was found that three of the randomized control trials showed no significant difference in clinical outcomes between the telemedicine and standard care groups. One randomized control trial study showed that telemedicine significantly improved disease remission, functional activity, and radiographic joint damage progression. Two of the studies showed no significant difference in patient satisfaction. It was concluded that there is no significant difference in patient-reported outcomes and patient satisfaction between the telemedicine and standard-of-care groups.^31^

Through an IRB-exempt retrospective review and additional interviews, a group of researchers investigated the overall outcomes, and provider and patient satisfaction with tele-rheumatology services that were provided by a medical centre. They found that inflammatory arthritis cases comprised the overwhelming majority (63.9% of all cases). It also became apparent that this visit type was not appropriate for 19% of the patients, due to disease complexity or unclear diagnoses. The authors concluded that while tele rheumatology services are successful at boosting the specialty care access of the rural populations, a triage system needs to be implemented to pair patients with the appropriate type of visit.^32^

Another study compared the effectiveness of virtual video-assisted visits in following up tightly with patients with inflammatory rheumatic diseases while taking face-to-face visits as their reference. Video visits were shown to be highly sensitive (94.1%) and specific (96.7%) in determining a need for treatment adjustment. On the other hand, they had low sensitivity at 55.6% in establishing the necessity for treatment tapering, especially in SLE patients. In addition, with the virtual video consultations, the patient-reported outcomes were reasonably reliable, but the disease activity measuring results were inconsistent.^33^

Between March and May 2020, during the COVID-19 lockdown, Thiele et al. investigated the outcomes of telemedicine visits for patients with rheumatoid arthritis, spondyloarthritis, or psoriatic arthropathy, whose in-person appointments were cancelled due to the pandemic. They report that through telemedicine visits, 17% of the patients had their treatments modified. Overall patient satisfaction with these consultations was high (mean score 4.3/5), however, lower satisfaction rates were associated with more severe pain. They concluded telemedicine visits can be highly suitable for selected patients. In addition, vaccination rates should be improved as they are at 38.2% for pneumococci and 53.6% for influenza.^34^

Through post-satisfaction questionnaires and pre/post-tests, Jong et al. investigated the favourability of in-person clinic visits, videoconference consults, and email access to a rheumatologist for patients with rheumatologic diseases in three rural communities of Canada. Their study showed that the videoconference method received the most positive feedback due to better accessibility, and an effective knowledge transfer between the referring physicians and the rheumatologists.^35^

A Qualitative semi structured Interview Study explored 15 RA patients’ self-reported experiences with telehealth follow-up. The study found that RA patients had positive perceptions of the PRO-based telehealth follow-up regarding flexibility and efficiency. Yet the lack of face-to-face contact with health professionals was reported negatively. The study suggested more studies to better understand how telehealth follow-up could be blended into routine clinical practice.^36^

A crossover randomised study was applied to compare the impact of hybrid care modality and face-to-face consultations on 121 rheumatoid arthritis patients’ report outcomes between October 2020 and May 2022. It was found that patients had low disease activity and low Health Assessment Questionnaire Disability Index score indicating the absence of disability. The study concluded that the hybrid care modality was non-inferior to face-to-face consultations in achieving patient-reported low disease activity and absence of disability during the COVID-19 pandemic in RA patients.^37^

A systematic review of 20 tele-rheumatology studies collected data from MEDLINE, Embase, Web of Science, and Scopus databases on the use of telemedicine. The cut-off date of the review was July 2015. The descriptive analysis reported that 18 out of 20 studies demonstrated positive results on the use of telemedicine. These 20 studies included 42% of RA patients. All six studies that included cost analysis found telemedicine to be cost-effective. The systematic review concluded that although the use of telemedicine is beneficial in the diagnosis and management of rheumatic diseases, still more studies are needed to conclude the most effective uses of telemedicine.^38^

In an observational retrospective longitudinal study, the impact of in-person and telemedicine healthcare delivery on rheumatoid arthritis (RA) and D2T RA patients were analysed respectively for three consecutive years (2019-2021). In 2019 in-person visit, in 2020 a combination of telemedicine and in-person visit, and 2021 in-person visit was applied for both groups. To compare the median of CDAI in difficult-to-treat (D2T) patients researchers applied quantile regression. The CDAI remission rate of RA patients was 40.55% (*N* = 163) in 2019, 43.18% (*N* = 155) in 2020 and 40.82% (*N* = 220) in 2021. The CDAI remission rate was also similar for the D2T patients across the three periods. was 22.22% (N=30); 23.68% (N=27); and 21.52% (N=34) respectively in 2019, 2020 and 2021. The study reported that percentages of D2T patients with a CDAI moderate/high disease activity were higher in 2019 (*N* = 63, 46.67%) than 2020 (*N* = 44, 38.6%) and 2021 (*N* = 55, 34.81%). The study results indicate that a combination of strategies (both in-person and telemedicine) that were used across the pandemic era was the most effective in treating RA and even D2T patients.^39^

In a descriptive cohort study during 2020 the level of therapeutic adherence, quality of life and self-care capacity of 71 RA patients were measured regarding telehealth. A univariate and bivariate analysis was performed (p-value <0.05). There was no statistically significant change in the quality of life and adherence to treatment as they remained the same. The self-care capacity improved significantly in five dimensions (*p* < 0.05). The study concluded that the application of telemedicine in treating RA patients gave the same baseline values in quality of life, adherence, and self-care capacity levels with in-person visits.^40^

In a cross-sectional survey, using short message service (SMS) and postal approaches a study was conducted on 2024 RA patients in England to analyse digital access, health and digital literacy, and impact on confidence and satisfaction with telemedicine consultations in RA patients. The data was collected from the DIAgnostic and MONitoring Database (DIAMOND) at Midlands Partnership NHS Foundation Trust in August 2021. Of 639 patients ([mean (s.d.) age 64.5 years) completed the survey, patients with lack of digital access (219), no internet use (93) and limited health or digital literacy (117) reported lower confidence and less satisfaction with telemedicine (both video and phone) consultation. The mean age of patients with no access to internet was 73.2 years. The study also reported that older age and not being employed increase challenge and therefore decrease confidence and satisfaction with telemedicine consultations. Clinical service providers should consider patients who are unable to access digital care and telemedicine and continue to provide in-person care services.^41^

A survey in a rheumatology outpatient clinic of the University Hospital Erlangen aimed to examine mobile Health (mhealth) usage, preferences, barriers, and eHealth literacy of German patients with rheumatic diseases. The survey was conducted between December 2018 and January 2019. 193 survey respondents’ self-reported data entry was analyzed. Mean age was 52.1 (SD 13.7) years. 34.7% (67) was 60 years and older and 53.9% (104) had been diagnosed with RA. It was reported that although most patients (188; 97.4%) are willing to use digital technologies in receiving health care and eager to use medical app, still patients’ eHealth literacy was found rather poor, and negatively correlated with patients’ age. Also, some patients’ preference of personal contact in receiving health care and concerns about data storage and transfer were reported among the findings.^42^

## DISCUSSION

A growing body of clinical studies show that telemedicine is beneficial and efficient in the management of RA patients and disease follow-up. Overall, studies found no statistically significant difference between RA patients on telemedicine and those who had in office visits. Patient-reported outcomes indicate positive perceptions of telemedicine and opportunities. More RA patients are satisfied with telemedicine; considered it flexible, convenient, effective, and efficient in terms of saving time and money and are inclined to use it in the future. The application of telemedicine in the intense monitoring of RA patients was also found beneficial. Video telemedicine is reported as a viable alternative for medical care for rheumatoid arthritis (RA) patients who have high disease activity and have positive perceptions of telemedicine, and for rheumatologists who regularly use telemedicine. The hybrid care modality was found noninferior to face-to-face consultations in achieving patient-reported low disease activity during the COVID-19 pandemic.

Nevertheless, not every health problem experienced by RA patients can be fully attended to via telemedicine. Lack of face-to-face contact with health professionals was reported negatively in some studies. Older age and lack of access to the internet, limited health and digital literacy decrease confidence and satisfaction with telemedicine. Telemedicine consultations were found to be a significant risk factor for no treatment changes in some studies. A triage system needs to be implemented to pair patients with the appropriate type of visit. More clinical studies are needed to better understand the best uses and the role of telemedicine for the management and quality of care for RA. Further research on how to improve and integrate telemedicine into routine clinical practice will be helpful. Large trials are needed to better assess the effects of web-based rehabilitation interventions in patients with rheumatoid arthritis. The study design, such as the observational nature and the unique setting of the previous research findings may be prone to bias including confounding, therefore may limit the generalisation of results. Evaluation in various settings and more randomised clinical studies are recommended. Cost, training, and technology requirements are also some challenges in the implementation of telemedicine. More future comparative studies on telemedicine vs standard care that investigate disease activity over time and quality of care for RA are needed. More studies are suggested on how telehealth follow-up could be blended into routine clinical practice.

More future research is required to understand the effectiveness of different tele-rheumatology interventions and various modes of telemedicine delivery in RA management. Also, more well-designed studies are needed to pinpoint potential limiting factors on RA patients’ access to telemedicine delivery. Factors such as data confidentiality and patient privacy issues,^9^ and inequities in accessing telemedicine care such as in RA patients in terms of geographic location, age, education, and socioeconomic status^43^ must be covered to better understand telemedicine in the management of RA patients. Clinical service providers should continue to provide in-person care services considering patients who have limited or no internet access.
